# Molecular Basis for the Dissociation Dynamics of Protein A-Immunoglobulin G1 Complex

**DOI:** 10.1371/journal.pone.0066935

**Published:** 2013-06-12

**Authors:** Fu-Feng Liu, Bo Huang, Xiao-Yan Dong, Yan Sun

**Affiliations:** Key Laboratory of Systems Bioengineering of the Ministry of Education and Department of Biochemical Engineering, School of Chemical Engineering and Technology, Tianjin University, Tianjin, China; University of Akron, United States of America

## Abstract

*Staphylococcus aureus* protein A (SpA) is the most popular affinity ligand for immunoglobulin G1 (IgG1). However, the molecular basis for the dissociation dynamics of SpA-IgG1 complex is unclear. Herein, coarse-grained (CG) molecular dynamics (MD) simulations with the Martini force field were used to study the dissociation dynamics of the complex. The CG-MD simulations were first verified by the agreement in the structural and interactional properties of SpA and human IgG1 (hIgG1) in the association process between the CG-MD and all-atom MD at different NaCl concentrations. Then, the CG-MD simulation studies focused on the molecular insight into the dissociation dynamics of SpA-hIgG1 complex at pH 3.0. It is found that there are four steps in the dissociation process of the complex. First, there is a slight conformational adjustment of helix II in SpA. This is followed by the phenomena that the electrostatic interactions provided by the three hot spots (Glu143, Arg146 and Lys154) of helix II of SpA break up, leading to the dissociation of helix II from the binding site of hIgG1. Subsequently, breakup of the hydrophobic interactions between helix I (Phe132, Tyr133 and His137) in SpA and hIgG1 occurs, resulting in the disengagement of helix I from its binding site of hIgG1. Finally, the non-specific interactions between SpA and hIgG1 decrease slowly till disappearance, leading to the complete dissociation of the SpA-hIgG1 complex. This work has revealed that CG-MD coupled with the Martini force field is an effective method for studying the dissociation dynamics of protein-protein complex.

## Introduction

*Staphylococcus aureus* protein A (SpA) is one of the most popular affinity ligands for immunoglobulin G (IgG) purification. Protein A affinity chromatography has been used as the industrial standard for IgG purification [1]. Nevertheless, Protein A affinity chromatography involves the following two critical challenges. Firstly, SpA is highly expensive and tends to lose activity as a result of harsh elution and washing conditions. Secondly, leaching of SpA may cause harmful immunogenic responses in humans. To address these issues and to realize bionic design of new affinity ligands, it is necessary to understand the molecular mechanism of the affinity interactions between SpA and IgG. At present, it is difficult to elucidate the molecular details by the available experimental approaches, so molecular simulations are considered useful to address the concerns by complementing experimental data.

Up to now, molecular simulations have been carried out to investigate the interactions between IgG and its ligands [2]–[4]. In particular, molecular dynamics (MD) simulations have proven to be a powerful tool in the study of protein-protein interactions [5], [6]. For example, some MD simulations have been carried out to investigate the interactions between IgG and some synthetic ligands [7], [8]. In addition, molecular mechanics-Poisson Boltzmann surface area (MM-PBSA) based on MD simulations was used to probe the molecular mechanism of the affinity between SpA and human IgG1 (hIgG1) [9]. The hot spots and binding motif of SpA are identified by the computational efforts. Moreover, the molecular basis for the effects of slat and pH on the affinity between SpA and hIgG1 has also been explored [10]. It revealed that the compensations between helices I and II of SpA as well as between the nonpolar and electrostatic energies made the binding free energy independent of salt concentration. However, at pH 3.0, the unfavorable electrostatic interactions increased greatly and became the driving force for the dissociation of the SpA-hIgG1 complex. Finally, the binding motif of SpA was modified based on the dissociation mechanism. However, the dissociation dynamics of SpA-hIgG1 complex is unknown at the molecular level. It is a difficult task to challenge by using all-atom MD simulations due to its enormous computational power. So, we have applied coarse-grained (CG) models to address this issue.

CG models use interactional sites that represent groups of atoms rather than explicitly including all of the atoms in a molecule [11]–[14]. So, CG models can reduce the number of simulated particles and allow much larger time steps [15]–[21]. It has been proven that CG-MD simulations can overcome the nanosecond timescale limitation of all-atom MD simulations to reach the biologically more relevant microsecond time scale [22]–[25]. CG-MD simulations have been successfully applied to explore the general theory of the protein folding [26]–[30], to study the protein-protein interactions [31]–[34], to investigate the interactions between lipid and protein [35]–[37], to probe the self-assembly of filled micelles on nanotubes [38], to explore the dynamics of four RNA-dependent RNA polymerases. Recently, many kinds of CG force fields have been built [39]. Of them, Martini is a widely used CG force field [15], [40]. It uses one interaction center for an average of four heavy atoms, so one to four beads are used for each amino acid. Despite lacking explicit polar hydrogen atoms, the Martini force field retains the polar and apolar nature of side chains of residues and their interactions in the coarse-grained representation of proteins [41].

Herein, the dissociation dynamics of SpA-hIgG1 complex was explored using CG-MD simulations coupled with the Martini force field. Firstly, CG-MD simulations were used to study the association of SpA-hIgG1 in water and different NaCl solutions. Some structural properties of SpA-hIgG1 complex (i.e., root mean square deviation of SpA and the fraction of the correct residues in the interface of the two proteins) and the intermolecular potential energies between SpA and hIgG1 were calculated to validate the feasibility of the CG-MD simulations with the Martini force field. Then, the potential energy contribution of each hot spot of SpA and hIgG1 was calculated and compared with the all-atom MD simulations. Finally, the dissociation dynamics of SpA-hIgG1 complex at pH 3.0 was explored to elucidate the molecular mechanisms by comparing the roles of the hot spots and the helices of SpA.

## Computational Methods

### Molecular Systems

**Figure 1:**
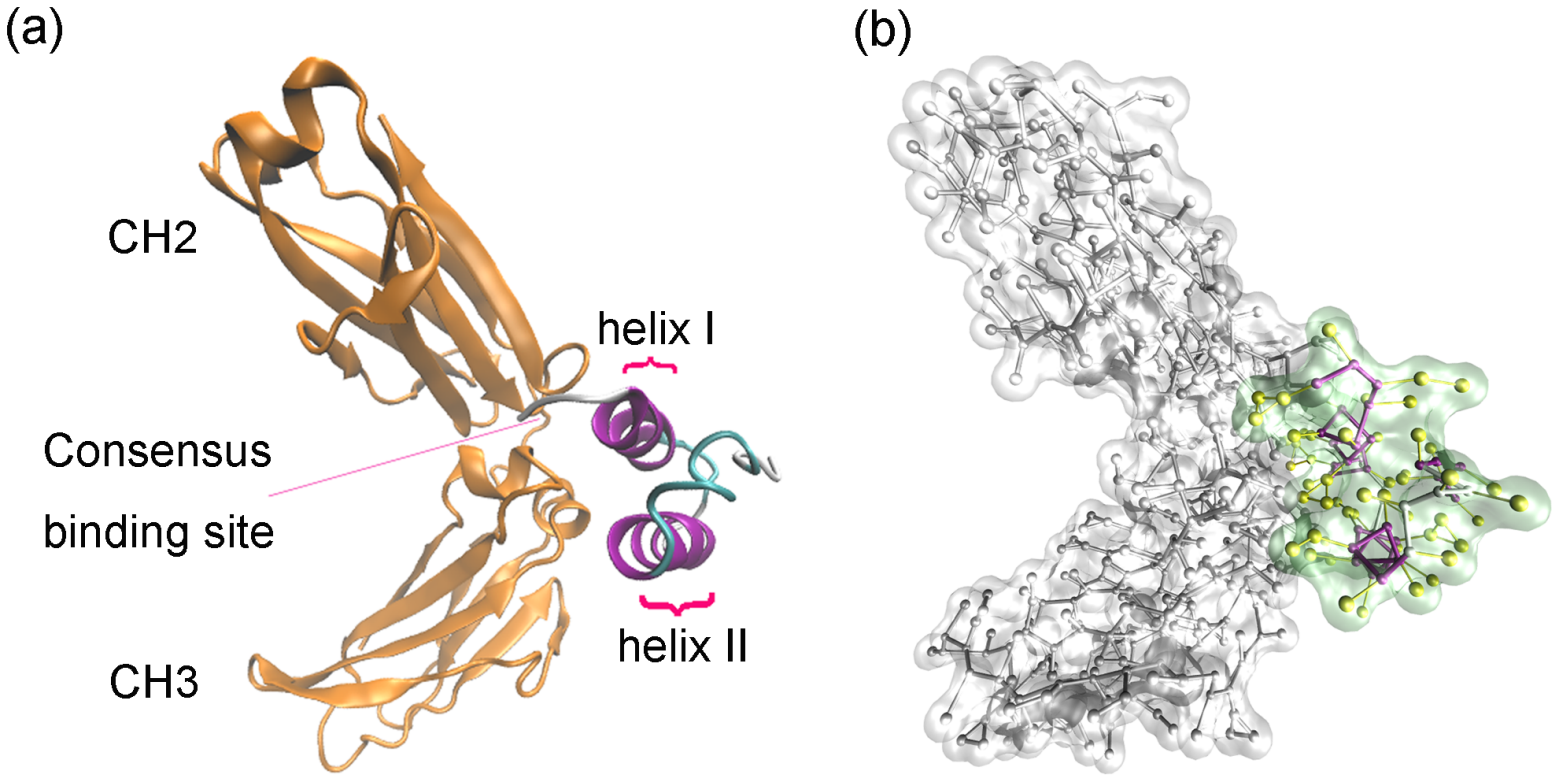
Molecular models of the SpA-hIgG1 complex. For clarity, the all atom model (a) is shown as Cartoon model. The Fc fragment of hIgG1 is colored by yellow and the binding parts of fragment B of SpA is colored according to its secondary structures: helix in purple and coil in blue. For the Martini coarse-grained model (b), the Fc fragment of hIgG1 is depicted by the surface and CPK model in white. Fragment B of SpA is also displayed by the surface and yellow CPK model. The α-helices are colored in purple and the coil is colored in white. The side chains of hIgG1 and SpA are colored in white and yellow, respectively.

Five systems with different concentrations of NaCl or at different pH were built for CG-MD simulations (Table 1). Each molecular system contains a single heavy chain of the Fc fragment of hIgG1 and the B domain of SpA (Figure 1). SpA model studied here ranges from Phe124 to Ala167 in sequence and consists of three α-helices connected by two irregular coils. For clarity, the helix from Lys126 to His137 and the helix from Glu144 to Asp155 are labeled as helices I and II, respectively [Figure 1(a)]. The all-atomic coordinates of the proteins were obtained from the X-ray structure in Protein Data Bank (PDB ID: 1FC2) [42], which includes 3997 atoms. The CG coordinates and the corresponding topology files of the two proteins were generated using atom2cg_v2.1.awk and seq2itp.pl scripts, respectively. These scripts are obtained from the Martini website (http://md.chem.rug.nl/cgmartini/index.php/downloads/tools/107-atom2cg). Figure 1b shows the Martini CG model of SpA-hIgG1 complex, which includes only 577 coarse grains. Water was represented by the CG water model. In this model, one single Martini water model represents four water molecules [15]. The hIgG1-SpA complex was solvated in a box using Martini water model. The size of the cubic box throughout the simulations was 8 nm with negligible volume ﬂuctuations. The different pH was mimicked by different protonation states of the charged residues [43], [44]. For all simulations at pH 7.0 and pH 3.0, the N termini and basic residues (Lys and Arg) were protonated and the C termini were deprotonated. The acidic residues (Asp and Glu) were deprotonated at pH 3.0 and pH 7.0. Residue His was protonated at pH 3.0 and kept neutral at pH 7.0. Na^+^ and Cl^−^ as salt ions were added according to the corresponding concentrations of NaCl and the charges of the proteins (Table 1).

**Table 1:** Summary of simulation systems.

System	N_Cl_^−^	N_Na_^+^	N_water_	Simulationlength (ns)^a^
pH 7.0, water	0	1	2389	500 (10)
pH 7.0, 0.25 mol/L NaCl	77	78	2235	500 (10)
pH 7.0, 0.5 mol/L NaCl	154	155	2081	500 (10)
pH 7.0, 1.0 mol/L NaCl	308	309	1773	500 (10)
pH 3.0, water	34	0	2381	500 (10)

a The number of repetitive simulation runs is given in parentheses.

### Coarse-grained Molecular Dynamics Simulation

All CG-MD simulations described in this paper were performed using GROMACS 4.0.5 package [45] with Martini force field [15], [41]. In order to mimic structural and dynamical properties of SpA-hIgG1 complex, an elastic network was used as a structural scaffold to describe and maintain the overall of the two proteins [46]. After minimization, the system was further equilibrated for 10 ns under an isothermal-isobaric (NPT) ensemble. The temperature (300 K) was kept constant using the Berendsen temperature weak coupling algorithm [47] with a coupling time constant of 1.0 ps. Isotropic pressure coupling was applied using the Berendsen algorithm with a reference pressure of 1 bar. A coupling constant of 5.0 ps and a compressibility of 4.5×10^−5^ bar^−1^ were used. Finally, production MD simulations were performed under isochoric isothermal (NVT) ensemble. The Lennard-Jones interactions were smoothly shifted to zero between 0.9 and 1.2 nm, using the switch potential implemented in GROMACS 4.0.5. The electrostatic interactions were shifted between 0 and 1.2 nm. Both energy and force vanish at the cutoff distance. The neighbor list was updated every 5 steps. The integration time step was 10 fs. For all simulations, the atomic coordinates were saved every 0.5 ns for analysis. To make certain that the dissociation of SpA-hIgG1 complex is intrinsic character rather than the stochastic output of MD simulations; ten MD simulations of 500 ns were conducted for each system under different initial conditions by assigning different initial velocities on each atom of the simulation systems (Table 1).

### Data Analysis

The simulation trajectories were analyzed using several auxiliary programs provided with the GROMACS 4.0.5 package. The programs include g_rms for calculating the root mean square deviation (RMSD) of the SpA-hIgG1 complex as a function of time and g_mindist for the number of contacts between the hIgG1 and SpA. The inter-molecular potential energies of SpA-hIgG1 complexes are calculated using CG-MD simulations with the Martini force field. The programs g_energy and make_ndx provided with the GROMACS 4.0.5 package are coupled to calculate the inter-molecular potential energies of the SpA-hIgG1 complex. The simulation data plotted in these figures are averaged over ten simulation trajectories, except those for the snapshots, which are plotted using the visual molecular dynamics (VMD) software (http://www.ks.uiuc.edu/Research/vmd/) [48].

## Results and Discussion

### Association of SpA-hIgG1 Complex

The Martini force field is one of the widely used coarse-grained force field of biomolecular dynamics simulations. Based on the various thermodynamic, dynamic and structural data, the Martini force field was parameterized in a systematic way. Extensive comparisons of the performance of the Martini model with respect to a variety of experimental properties has revealed that the model performs generally quite well (“semi-quantitatively”) for protein systems. Properties accurately reproduced include structural, dynamic and thermodynamic data. Therefore, the Martini force field was sufficiently tested. In addition, the transferability and predictability of the Martini force field has been proven [49]. That is, the Martini force field can be used to study protein systems without requiring further reparametrization. Moreover, the Martini force field has been used to study a wide range of chemical systems without requiring further reparametrization. For example, the Martini force field has been successfully applied in numerous studies of protein-membrane interactions [50]–[53] and the protein-protein association [54]–[56]. Herein, the Martini force field was used to probe the molecular mechanism of the dissociation dynamics of SpA-hIgG1 complex. Firstly, it is necessary to validate whether the Martini force field can reproduce the association process of SpA-hIgG1 complex? So, the initial X-ray structure of SpA-hIgG1 complex is broke up and the distance between SpA and hIgG1 is about 5 Å, which is used as the initial structure in the association simulations.

**Figure 2:**
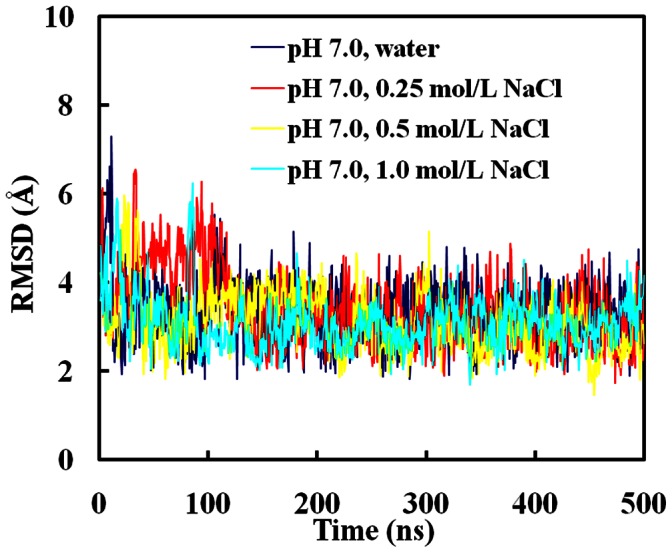
The root mean square deviation (RMSD) of SpA for one trajectory as a function of time for simulations in water and different NaCl solutions at pH 7.0.

**Figure 3:**
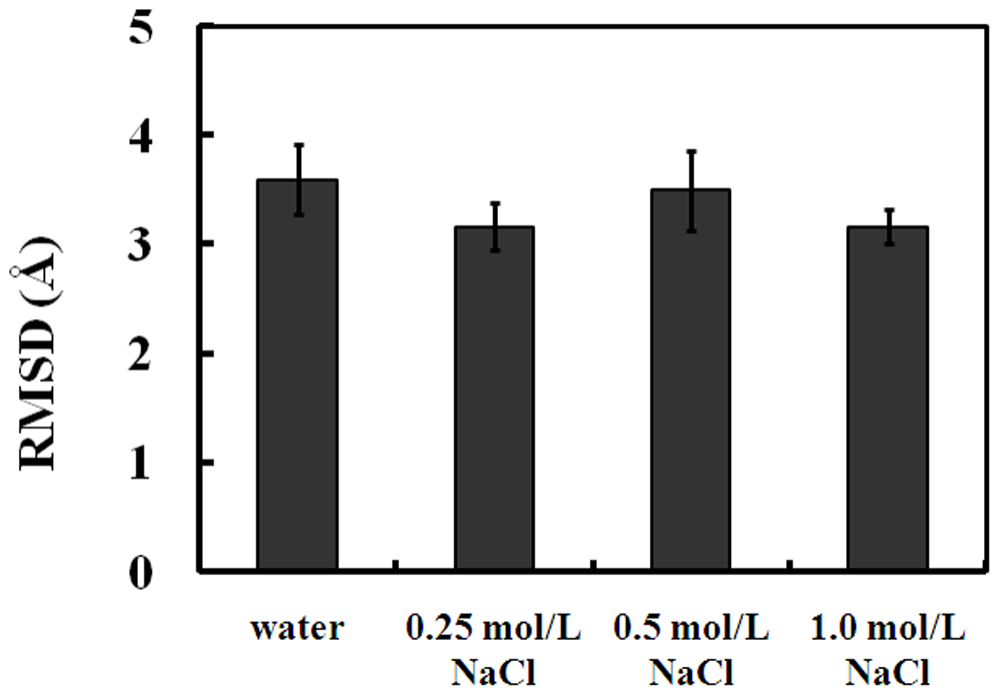
The root mean square of deviation (RMSD) (average of all ten parallel simulations in the same condition) for SpA along the last 100 ns trajectory in water and different NaCl solutions at pH 7.0.

RMSD was used to represent the conformational changes of SpA in water and different NaCl solutions. Figure 2 shows the RMSD profile of SpA for one trajectory as a function of time for simulations in water and different NaCl solutions at pH 7.0. It can be seen that the RMSD values of SpA decrease greatly in 10 ns and approach stable values (∼ 3 Å) in water and different NaCl solutions. It indicates that SpA binds rapidly to hIgG1 and forms a stable complex in water and different NaCl solutions. The averaged RMSD values of SpA along the last 100 ns trajectory in water and different NaCl solutions at pH 7.0 are shown in Figure 3. It indicates that the salt almost does not affect the conformation of SpA-hIgG1 complex in NaCl solutions ranging from 0 to 1.0 mol/L. That is, NaCl cannot disrupt the affinity between SpA and hIgG1, which agrees well with the experimental results and our previous all-atom MD simulations [10], [57].

**Figure 4:**
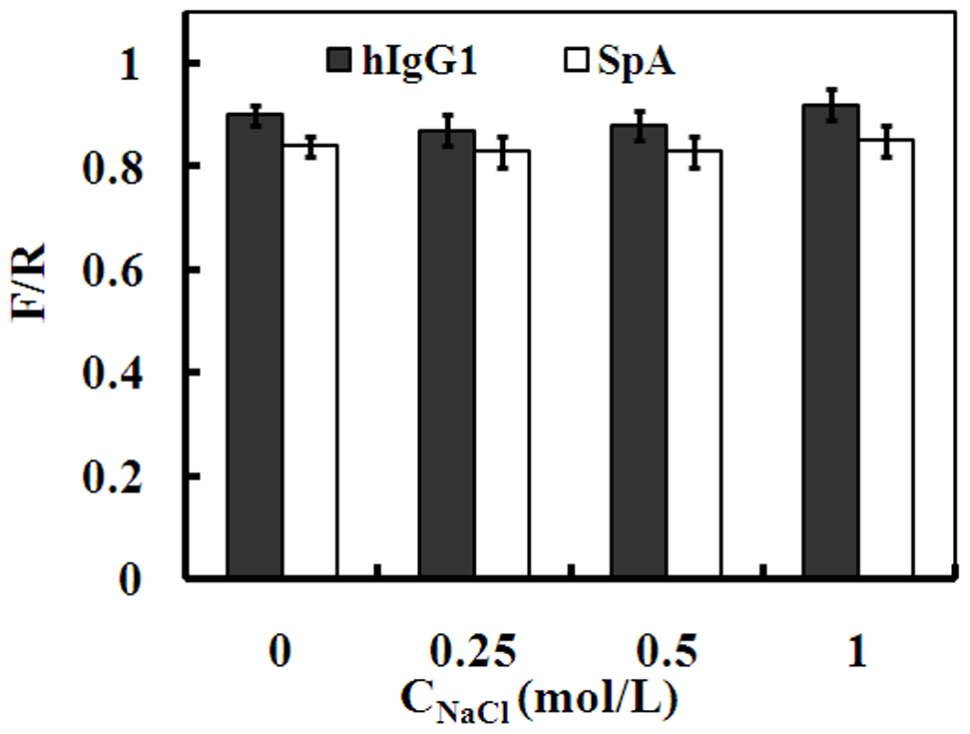
Fraction of the correct residues (F/R) in the interface of SpA and hIgG1 (average of all ten parallel simulations in the same condition) in water and different NaCl solutions at pH 7.0.

In order to probe the effect of NaCl on the conformations of SpA-hIgG1 complex, the fraction of the correct residues in the interface (F/R) was also used to characterize the binding interface of SpA-hIgG1 complex. F/R is defined as the number of the correct residues in the interface divided by the total number of the residues in the interface [58]. In this study, the correct residues are defined as the residues within the interface using the stable SpA-hIgG1 complex in water. In addition, the residues in the interface are defined using the cutoff distance between the partner proteins of 10 Å. In different salt solutions, the values of F/R of SpA and hIgG1 are similar with those in water (Figure 4). It means that SpA interacts with the consensus-binding site of hIgG1, and the salt almost does not affect the affinity between SpA and hIgG1, which are also consistent with the previous results of all-atom MD simulations [10]. In addition, the values of F/R of SpA are about 0.85 in water and different salt solutions (Figure 4), which are slightly lower than those of hIgG1 (0.90). It is caused by the flexibility of the coil of SpA because that it cannot bind closely to the binding site of hIgG1 during the binding process of SpA-hIgG1 complex.

**Figure 5:**
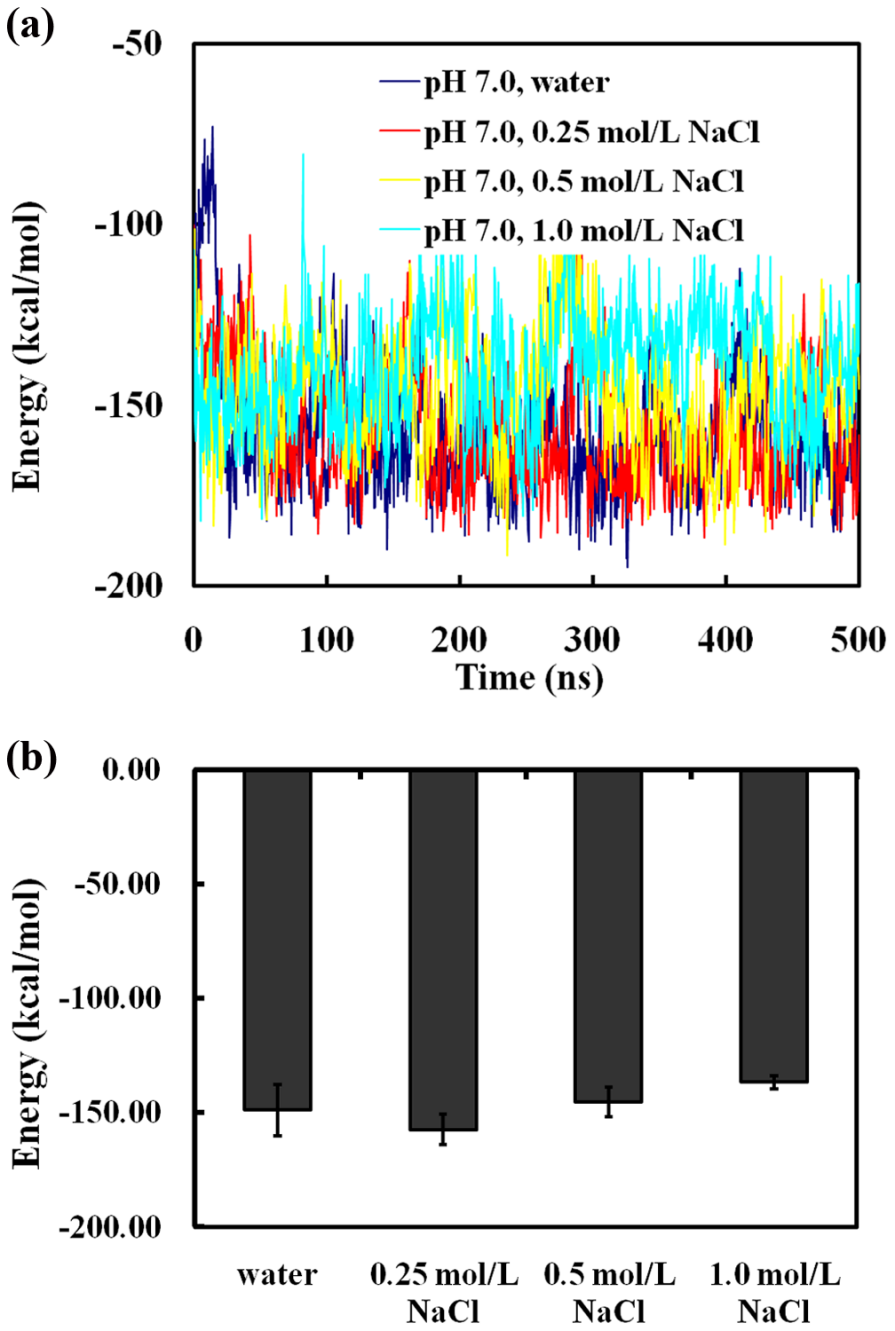
Inter-molecular potential energy analysis of the SpA-hIgG1 complex. (a) Inter-molecular potential energy as a function of time for the simulations in water and different NaCl solutions at pH 7.0. (b) Average intermolecular potential energies (average of all ten parallel simulations in the same condition) of the SpA-hIgG1 complex along the last 100 ns trajectory in water and different NaCl solutions at pH 7.0.

The intermolecular potential energies of SpA-hIgG1 complex in water and different NaCl solutions are shown in Figure 5. Figure 5(a) shows the intermolecular energies of SpA-hIgG1 complex as a function of time for the simulations in water and different NaCl solutions at pH 7.0. It is obvious that the energies of the SpA-hIgG1 complex decrease rapidly at the initial 10 ns, and they are all essentially approach stable values (∼150 kcal/mol) after 50 ns. In addition, the average intermolecular potential energies of the SpA-hIgG1 complex are similar (∼ −150 kcal/mol) along the last 100 ns trajectory in water and different NaCl solutions [Figure 5(b)]. It further indicates that NaCl does not affect the affinity between SpA and hIgG1.

From our previous simulation results [9], [10], the affinity between SpA and hIgG1 is mainly dominated by a couple of hot spots. In order to further validate the creditability of CG-MD simulations with the Martini force field, inter-molecular potential energy contribution of each residue of SpA and hIgG1 in SpA-hIgG1 complex was calculated to find the contributions of the residues and compared with the results of all-atom simulations. Herein, the criterion of 2.5 kcal/mol is also used to identify the hot spots.

**Figure 6:**
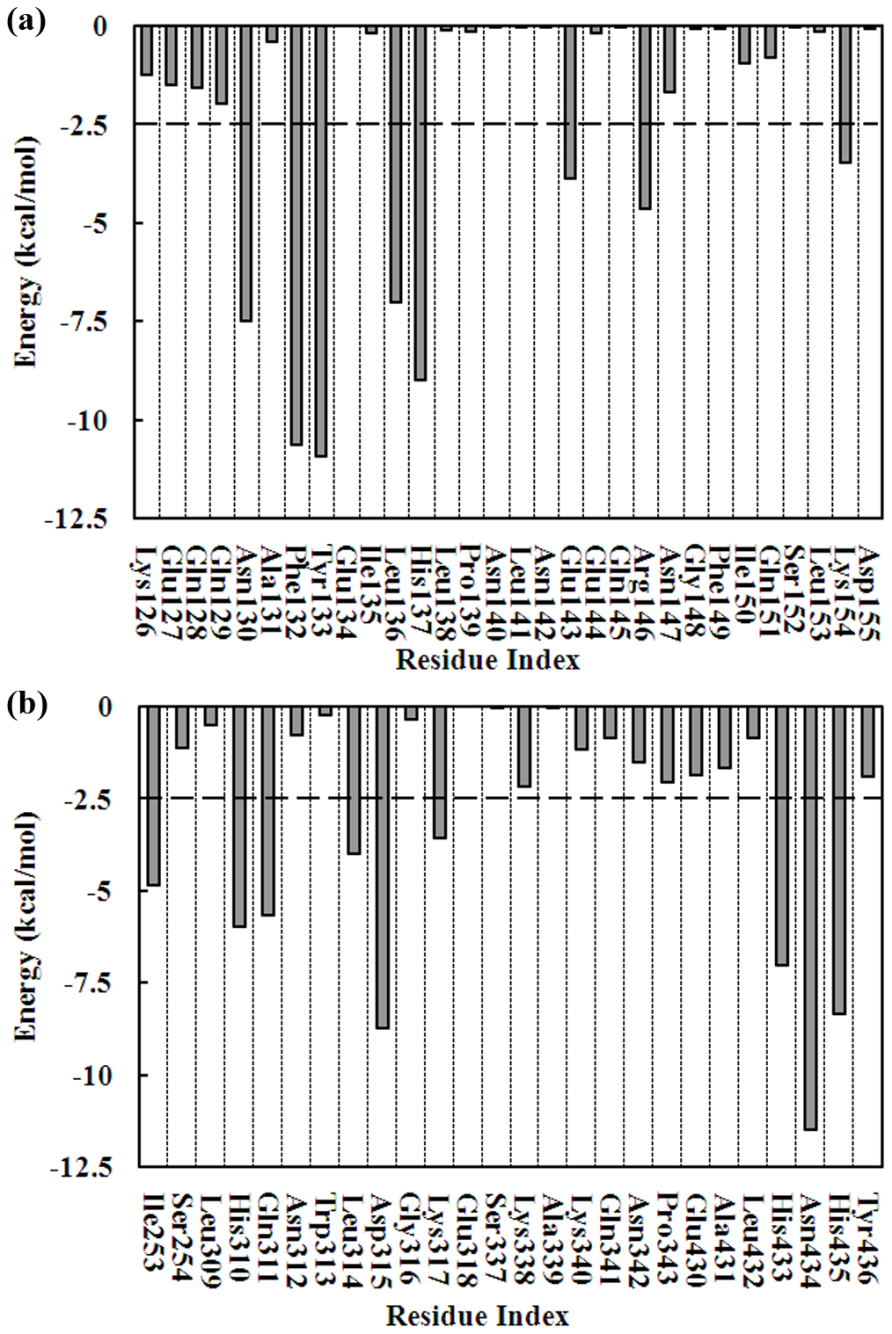
Inter-molecular potential energy contribution of some residues of the SpA-hIgG1 complex in water at pH 7.0. (a) SpA and (b) hIgG1.

Figure 6 shows the inter-molecular potential energy contribution of some residues of SpA-hIgG1 complex in water at pH 7.0. It reveals that, as for SpA, eight residues (i.e., Asn130, Phe132, Tyr133, Leu136, His137, Glu143, Arg146, and Lys154) make great contribution, while the other residues have minimal contributions [Figure 6(a)]. Of them, six residues (i.e., Phe132, Tyr133, His137, Glu143, Arg146 and Lys154) are consistent with the results of all-atom simulations [9], [10]. On the contrary, the inter-molecular potential energies of the other two residues (Asn130 and Leu136) are magnified compared with the results of all-atom MD simulations. Figure 6(b) shows the potential energy contribution of some residues for hIgG1 in water at pH 7.0. From Figure 6(b), 9 residues (i.e., Ile253, His310, Gln311, Leu314, Asp315, Lys317, His433, Asn434, and His435) provide less than −2.5 kcal/mol. Six of them (i.e., Ile253, His310, Gln311, Asp315, Lys317, and Asn434) are in agreement with the results of all-atom simulations [9]. In contrast, the three residues (i.e., Leu314, His433 and His435) are also magnified by the Martini force field.

The inter-molecular potential energy contribution of some residues of SpA-hIgG1 complex in different (0.25, 0.5 and 1.0 mol/L) NaCl solutions was also calculated and listed in Figures S1–3. It is clear that most of hot spots (i.e., Phe132, Tyr133, His137, Glu143, and Arg146) make great contribution in 0.25–1.0 mol/L NaCl solutions. However, the inter-molecular potential energy of residue Lys154 is greatly affected by the salt. At lower NaCl concentrations (0.25 and 0.5 mol/L), residue Lys154 has the minor contribution, which is below the criterion of −2.5 kcal/mol [Figures S1–2 (a)]. On the contrary, residue Lys154 provides about −3.3 kcal/mol in 1.0 mol/L NaCl solution [Figure S3 (a)]. It is noted that residues Asn130 and Leu136 are also magnified by the Martini force field, which agrees well with that in water. In addition, in NaCl solutions, residue Gln129 is magnified by the Martini force field. Figures S1–3 (b) shows the inter-molecular potential energy contribution of some residues of hIgG1 in different NaCl solutions. It is shown that most of hot spots (i.e., Ile253, Gln311, Asp315, Lys317, and Asn434) make great contribution in different (0.25–1.0 mol/L) NaCl solutions. However, the energies provided by residue His310 are below the criterion of −2.5 kcal/mol in 0.5 mol/L NaCl solution. Moreover, residues Leu314, His433 and His435 are also magnified by the Martini force field, which is consistent with that in water.

The magnification of the potential energies is mainly caused by the Martini force field. It is known that the non-bond interactions among the Martini particles are mainly described using a shifted Lennard-Jones 12-6 potential energy function [15]. It is known that the strength of the interactions increases with the increasement of both the distance and the contact area between two these residues because that the Lennard-Jones force is a kind of short-range force. From Figure 1, it is clear that some residues around the hot spots of SpA have more chance to get close to the residues of hIgG1 and have more contact areas. For example, residues Asn130 and Leu136 of SpA locate around the hot spots Phe132 and His137, respectively. As for Fc fragment of hIgG1, residues His433 and His435 distribute around the hot spot Asn434. And, residue Leu314 locates near the hot spot Asp315. Therefore, the potential energies provided by these residues are magnified. Moreover, the magnification of the potential energies using Martini force field is also found in our previous study [59]. It is found that the standard Martini force field generates too strong adsorption of lysozyme on the agarose matrix and ligands of hydrophobic charge induction chromatography. Therefore, CG-MD simulations with the Martini force field can identify all of the hot spots of SpA-hIgG1 complex although it can magnify the potential energies of the two residues of SpA (Asn130 and Leu136) and the three residues of hIgG1 (Leu314, His433 and His435).

The association of SpA-hIgG1 in water and different NaCl solutions was studied by CG-MD simulations with the Martini force field. The structural properties (i.e., RMSD of SpA and F/R of SpA and hIgG1 complex) and the inter-molecular potential energies obtained using CG-MD simulations indicate that the affinity between SpA and hIgG1 is affected little by NaCl at pH 7.0. These results are consistent with many experimental studies [1], [60]. In addition, the hot spots of SpA and hIgG1 are also identified using CG-MD simulations with the Martini force field, which is remarkably consistent with our previous all-atom MD simulations. In addition, the accuracy of the CG-MD simulations is often established via comparison with the equivalent all-atom simulations, which has been widely used in literature [56], [61], [62]. Therefore, CG-MD simulations with the Martini force field are feasible to study the dynamics properties of SpA-hIgG1 complex although the potential energies of some residues are magnified using Martini force field.

### Dissociation Dynamics of SpA-hIgG1 Complex

Our previous all-atom MD simulations have proven that there are repulsive interactions between SpA and hIgG1 at pH 3.0 [10]. But the 70 ns all-atom MD simulations cannot produce the dissociation dynamics of SpA-hIgG1 complex. Herein, CG-MD simulations with the Martini force field were used to probe the dissociation dynamics of SpA-hIgG1 complex at pH 3.0.

**Figure 7:**
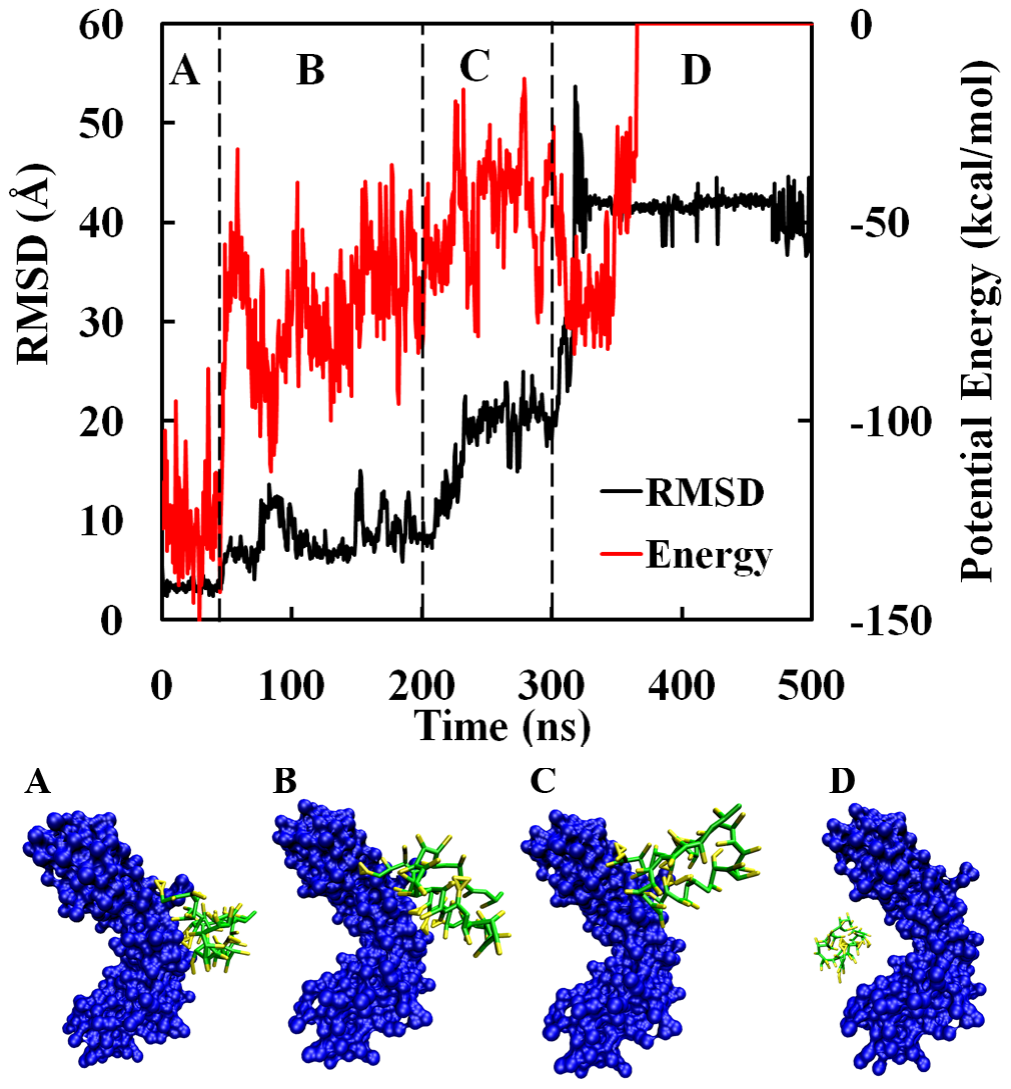
Time-development of the root mean square deviation (RMSD) and inter-molecular potential energy of SpA-hIgG1 complex and the corresponding representative snapshots of SpA-hIgG1 complex along the trajectory in water at pH 3.0. (A) 28 ns; (B) 170 ns; (C) 280 ns; (D) 436 ns. The Fc fragment of hIgG1 is depicted by the surface model in blue. The fragment B of SpA is displayed in Licorice model. The backbone and side chains of SpA are colored in green and yellow, respectively.

Starting from the stable SpA-hIgG1 complex, the dissociation dynamics was studied by CG-MD simulations with the Martini force field at pH 3.0, which were conducted 10 sets of 500 ns CG-MD simulations. It is noted that 500 ns is sufficient to produce the dissociation dynamics of SpA-hIgG1 complex. Moreover, similar features were observed in all sets of the CG-MD simulations, although each simulation has started from different unbiased initial conditions. First, two parameters, the RMSD of SpA from its initial structure and the intermolecular potential energy for SpA-hIgG1 complex, were used to represent the dissociation dynamics of SpA-hIgG1 complex. RMSD values of SpA and the intermolecular potential energy for the SpA-hIgG1 complex are displayed as a function of time in Figure 7. In addition, four corresponding snapshots of the SpA-hIgG1 complex at pH 3.0 are also shown. From Figure 7, it indicates that the RMSD and the inter-molecular potential energy of SpA-hIgG1 complex increase greatly along with the simulation time. It implies that the affinity between SpA and hIgG1 is destroyed gradually and the SpA-hIgG1 complex dissociated completely after 300 ns at pH 3.0. The phenomenon revealed by the CG-MD simulations is also consistent with the experimental results [57]. In addition, it is clear that there are four steps during the dissociation dynamics of SpA-hIgG1 complex. The first step is the conformational adjustment of SpA during the initial 40 ns. At this stage, SpA still binds to the consensus binding site of hIgG1 (Snapshot A in Figure 7). So, the values of RMSD show little change (Figure 7). On the contrary, the intermolecular potential energies for SpA-hIgG1 complex fluctuate from −150 to −100 kcal/mol. That is, the binding motif of SpA and hIgG1 is affected little during the initial 40 ns although the affinity between SpA and hIgG1 is greatly influenced at pH 3.0.

During 40–200 ns, the inter-molecular potential energies increase rapidly to about −50 kcal/mol and fluctuate greatly in the range from −50 to −70 kcal/mol. In contrast, the values of RMSD increase slowly to about 12 Å. It indicates that the affinity between SpA and hIgG1 is disrupted partly and some parts of SpA are dissociated from their binding sites of hIgG1. Meanwhile, SpA moves slowly from its binding site of hIgG1, which is shown as snapshot B in Figure 7. Then, potential energies continue increasing slowly and the values of RMSD increase rapidly during 200–300 ns. From the snapshot C in Figure 7, it obviously shows that the two α-helices of SpA move away from their binding sites of hIgG1. It means that the affinity between SpA and hIgG1 is completely broken. However, there are some strong non-specific interactions between SpA and other parts of hIgG1 (Snapshot C in Figure 7), which is the reason of the slow increasing of potential energies and high increasing of RMSD values. After 300 ns, the non-specific interactions decrease slowly to zero, leading to the complete dissociation of the SpA-hIgG1 complex (Snapshot D in Figure 7).

**Figure 8:**
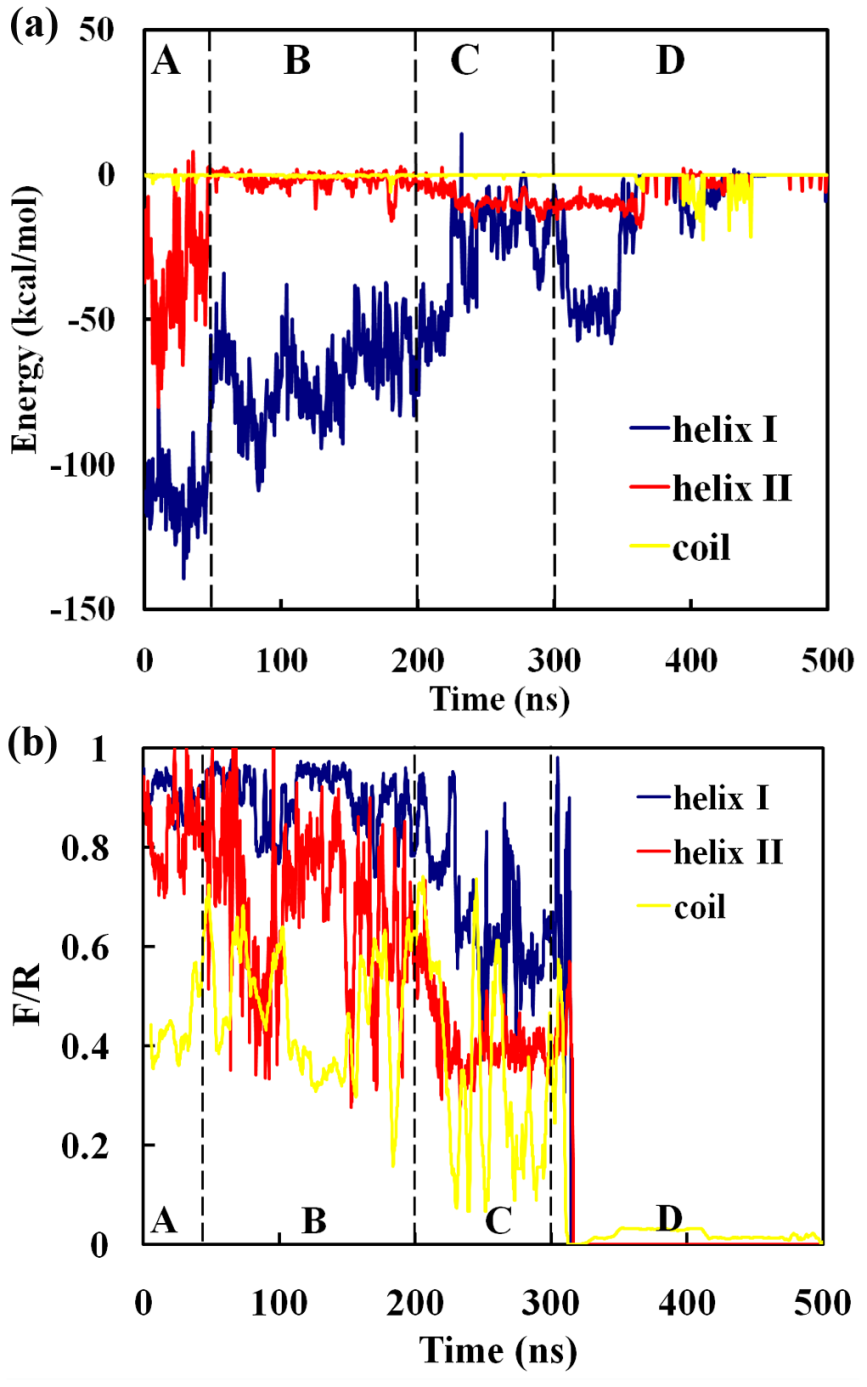
Potential energies and structural analysis of the three parts (i.e., helix I, helix II and coil) of SpA as a function of time in water at pH 3.0. (a) Potential energies; (b) The values of the fraction of these residues in the interface (F/R) of SpA and hIgG1.

From Figure 1, only two α-helices (I and II) and the random coil connecting them of SpA binds Fc of hIgG1. Our previous studies have revealed that helix I binds Fc through hydrophobic interactions, while helix II mainly provides electrostatic interactions [9], [10]. In order to probe the effect of the two α-helices and the coil during the dissociation process, two parameters (i.e., F/R and inter-molecular potential energies of the three parts of SpA) were used to probe their function in the dissociation process of SpA-hIgG1 complex at pH 3.0. The values of F/R and the inter-molecular potential energies of the three parts (i.e., the two α-helices and the coil) of SpA are displayed as a function of time in Figure 8. It can be seen from Figure 8(a) that the inter-molecular potential energy between the coil and hIgG1 is almost zero during the whole CG-MD simulations except that it provides some non-specific interactions at the fourth stage. It indicates that the coil almost does not affect the affinity between SpA and hIgG1, which is consistent with the previous results of the experiment and our all-atom MD simulations [9], [10], [42]. Thus, the coil of SpA cannot be considered in the following analysis.

Although the inter-molecular potential energy provided by helix II is less than that of helix I, it is greatly affected at pH 3.0 during the initial 40 ns. That is, the inter-molecular potential energy of helix II is weakened firstly and increases to zero after about first 40 ns [Figure 8(a)]. At pH 7.0, helix II of SpA and its corresponding binding site of hIgG1 carry −2e and +3e charges, respectively. So, there are strong favorable electrostatic interactions between helix II of SpA and hIgG1 at pH 7.0. At pH 3.0, however, all of the electrostatic residues (i.e., Glu143, Arg146 and Lys154) of SpA are positively charged or neutral. Meanwhile, the corresponding charged residues of hIgG1 (i.e., Arg255, Lys288, Lys317, Lys338, Asp249, Glu258, Asp280, Asp315, Glu333, Glu345 and Glu380) are also positively charged or neutral. So, at pH 3.0, SpA and hIgG1 carry +4 e and +30 e, respectively. Therefore, the electrostatic interactions between the three residues (i.e., Glu143, Arg146 and Lys154) of helix II in SpA and hIgG1 are disrupted completely at pH 3.0. That is, at pH 3.0, the favorable electrostatic interactions between these charged residues are destroyed. So, the potential energies dominated by helix II increase from −50 kcal/mol to zero after 40 ns [Figure 8(a)]. Meanwhile, the values of F/R of helix II fluctuate around 70%∼90%. It indicates that the conformation of helix II is greatly affected. On the contrary, the potential energies provided by helix I keep stable at about −120 kcal/mol [Figure 8(a)] and the values of F/R of helix I also keep stable around ∼90% [Figure 8(b)]. That is, the affinity between helix I and hIgG1 keeps stable during the first 40 ns.

After 40 ns, the potential energies of helix I increase sharply from its initial −120 to about −50 kcal/mol and fluctuate around it [Figure 8(a)]. In contrast, as shown in Figure 8(b), the F/R values of helix I decrease to about 80% during the second stage. It indicates that the affinity between helix I of SpA and hIgG1 is disrupted partly although it binds closely to its binding site of hIgG1 at this stage. However, the F/R values of helix II fluctuate greatly in the range from 30% to 100% although its potential energy is stable about zero. It indicates that the affinity between helix II of SpA and hIgG1 has been disrupted completely. Meanwhile, helix II of SpA has been dissociated from its binding site.

At the third stage (200–300 ns), the potential energies between helix I of SpA and hIgG1 increase gradually to about zero. Meanwhile, the values of F/R of helix I decrease greatly from its initial 95% to about 50%, which indicates that helix I moves gradually from its binding site and finally dissociates completely from its binding site of hIgG1. Therefore, the affinity between helix I of SpA and hIgG1 is completely destroyed at this stage. After 300 ns, the F/R values of the two helices decrease sharply to about zero. It indicates that SpA has been dissociated completely from its binding site of hIgG1. Meanwhile, inter-molecular potential energies provided by the helices I and II are almost zero although some week non-specific interactions exist between SpA and the other residues of hIgG1. For example, from 310 to 340 ns, the potential energies between helix I of SpA and hIgG1 are about −50 kcal/mol. They are provided by non-specific interactions because that the corresponding values of F/R of helix I are about zero. Therefore, the affinity between SpA and hIgG1 has been destroyed completely at the fourth stage.

**Figure 9:**
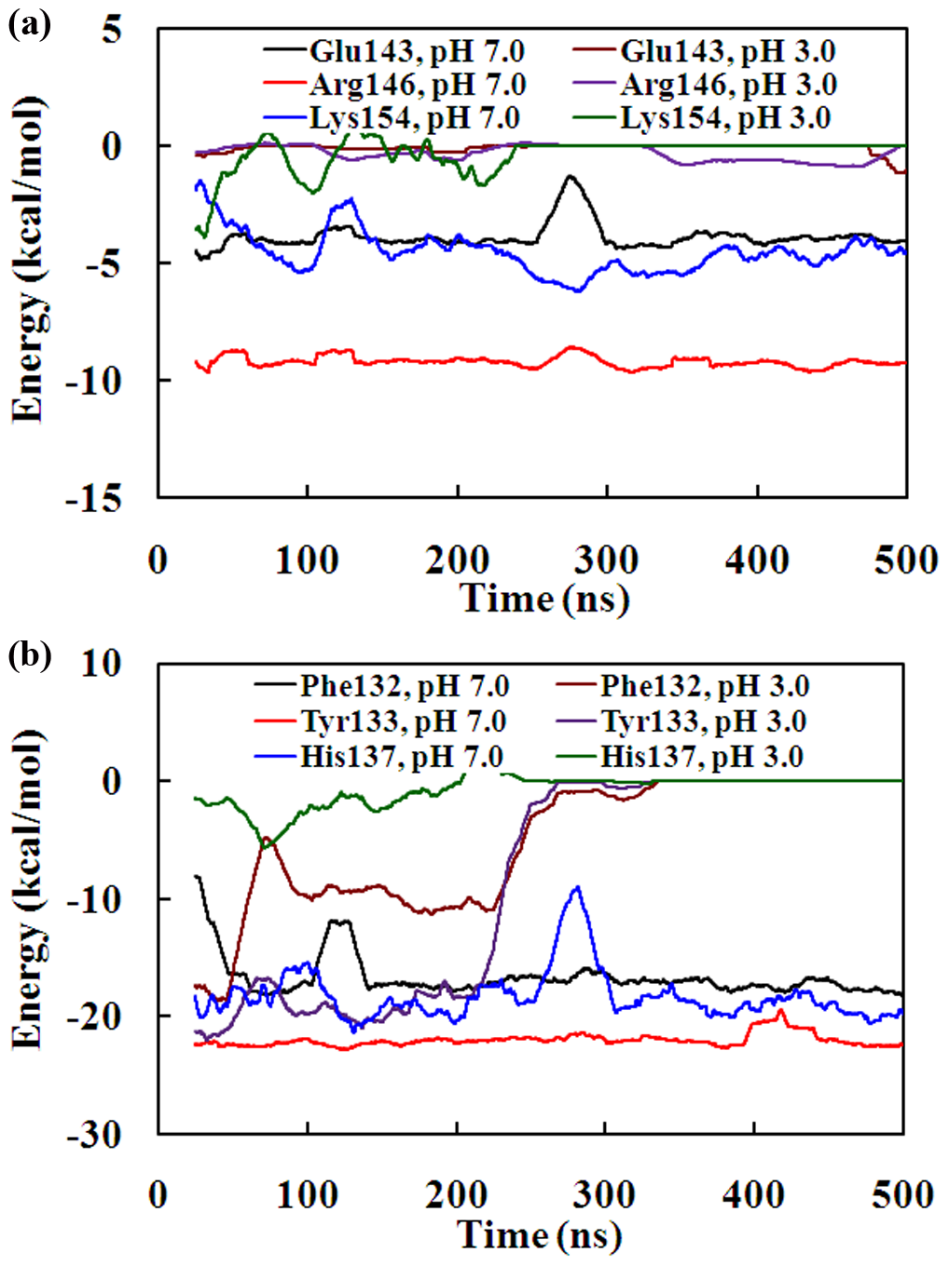
Potential energy contributions of the six hot spots of SpA as a function of time in water at pH 7.0 and pH 3.0, respectively. (a) The hot spots (Glu143, Arg146 and Lys154) of helix II in SpA; (b) The hot spots (Phe132, Tyr133 and His137) of helix I in SpA.

Previous all-atom MD simulations have proven that the affinity between SpA and hIgG1 are dominated by the six key residues (i.e., Phe132, Tyr133, His137, Glu143, Arg146 and Lys154) [9], [10]. However, the effect of these residues on the affinity between SpA and hIgG1 during the dissociation process remains unknown. So, the potential energies between these residues and hIgG1 were calculated at pH 7.0 and pH 3.0, respectively. From Figure 8, we know that the electrostatic interactions between helix II of SpA and hIgG1 are firstly affected at pH 3.0. So, the potential energies provided by the charged residues Glu143, Arg146 and Lys154 of helix II were calculated at pH 7.0 and pH 3.0, respectively. As shown in Figure 9(a), all of the residues offer favorable interactions for the affinity between SpA and hIgG1 at pH 7.0. For example, the residue Arg146 provides about −10 kcal/mol potential energy, which is lower than those of the other two hot spots (Glu143 and Lys154). However, the potential energies provided by both Arg146 and Lys154 change immediately to zero at pH 3.0 [Figure 9(a)]. The main reason is that the two residues and the corresponding residues of hIgG1, which interacts with the two residues, have positive charge at pH 3.0. For example, each of two hot spots (Arg146 and Lys154) has +1 e at pH 3.0. Meanwhile, the corresponding residues of hIgG1 carry about +30 e. So, there are some strong repulsive force between the residues and hIgG1 at pH 3.0. In addition, Glu43 is neutral state at pH 3.0. So, its potential energy increases slowly during the first 40 ns. And then, their potential energies fluctuate around zero during the second stage. Therefore, the electrostatic interactions of the three hot spots (i.e., Glu143, R146 and K154) of helix II of SpA are first disrupted. And then, helix II departs from the binding site of hIgG1 and moves gradually outside from its binding site of hIgG1 (Figure 8).

Figure 9(b) shows the potential energies provided by the hot spots (i.e., Phe132, Tyr133 and His137) of helix I at pH 7.0 and 3.0, respectively. It is obvious that about −20 kcal/mol potential energy is provided by every hot spot at pH 7.0. However, their interactions are affected differently at pH 3.0. Compared to the other two hydrophobic residues Phe132 and Tyr133, the interactions between residue His137 and hIgG1 is firstly alleviated at pH 3.0. From our previous all-atom MD simulations, His137 interacts with the residues Leu314, Asp315, His429 and Asn434 of hIgG1 via electrostatic interactions including hydrogen bonds at pH 7.0. At pH 3.0, however, residue His has +1e. So, there are some repulsive interactions between His137 of SpA and His429 of hIgG1. Then, the affinity between His137 of helix I and hIgG1 is firstly disrupted during the first stage. Therefore, the residue His137 also plays an important role in the dissociation dynamics process. On the contrary, the potential energies of the other two hydrophobic residues (Phe132 and Tyr133) are about −20 kcal/mol at the first stage, which are similar with those in pH 7.0 [Figure 9(b)]. That is, the affinity between the two residues (Phe132 and Tyr133) and hIgG1 is not affected at the first stage. After about 40 ns, the potential energies of Phe132 increase sharply from −18 to about −5 kcal/mol, and then decrease to −10 kcal/mol. It indicates that the affinity between Phe132 and hIgG1 is affected greatly from the second stage. And then, the potential energies of Phe132 increase gradually to zero from the third stage. Therefore, the interactions between Phe132 and hIgG1 are affected differently at different stages. In contrast, the potential energies provided by Tyr133 keep about −20 kcal/mol until 200 ns, which is consistent with that at pH 7.0. And then, the potential energies provided by Tyr133 increase greatly to about zero after 300 ns. It indicates that the affinity between the Tyr133 and hIgG1 is completely destroyed.

From the above analysis, it can be concluded that the dissociation of SpA-hIgG1 complex is mainly dominated by the four charged residues (i.e., His137, Glu143, Arg146 and Lys154). At pH 3.0, the electrostatic interactions of the three residues (i.e., Glu143, Arg146 and Lys154) are firstly disrupted at the first stage. So, helix II of SpA dissociated from its binding site. Under the influence of the dissociation of helix II, the potential energies provided by the residue His137 are disrupted. The hydrophobic interactions of Phe132 are then alleviated partly. Finally, the hydrophobic interactions provided by residues (i.e., Phe132 and Tyr133) are disrupted completely after 300 ns. Finally, helix I dissociates completely from its binding site of hIgG1. In other words, the four charged residues provide favorable interactions for the association of SpA and hIgG1 at pH 7.0. On the contrary, the affinity between these charged residues and hIgG1 is firstly disrupted at pH 3.0. In conclusion, the affinity between SpA and hIgG1 is regulated by these charged residues (i.e., His137, Glu143, Arg146 and Lys154) at pH 3.0.

### Conclusions

In this article, the dissociation dynamics of SpA-hIgG1 complex was studied using CG-MD simulations with the Martini force field. Firstly, the association of SpA-hIgG1 complex is studied by CG-MD simulations with the Martini force field in water and different salt solutions at pH 7.0. The results of structural properties (i.e., RMSD of SpA and F/R of SpA and hIgG1 complex) and the inter-molecular potential energy indicate that the affinity between SpA and hIgG1 is affected little by NaCl at pH 7.0. It is further confirmed that CG-MD simulations with the Martini force field can identify all of the hot spots, which is found in our previous all-atom MD simulations. It is verified that CG-MD simulations with the Martini force field can be used to study the association dynamics of SpA-hIgG1 complex. And then, the dissociation dynamics of SpA-hIgG1 complex was probed at pH 3.0 using CG-MD simulations with the Martini force field. It is found that there are four steps during the dissociation dynamics of SpA-hIgG1 complex. Firstly, the conformation of helix II of SpA is modulated. The electrostatic interactions between the three charged residues (Glu143, Arg146 and Lys154) of helix II and hIgG1 are disrupted. So, helix II of SpA moves gradually outside from the binding site of hIgG1. Subsequently, the affinity of residue His137 in helix I is also destroyed. Thereafter, the hydrophobic interactions between the hot spots (Phe132 and Tyr133) of helix I and hIgG1 are also disrupted and helix I of SpA moves away from its binding site of hIgG1. Finally, SpA dissociates completely from its binding site of hIgG1 after 300 ns. These CG-MD simulations provide direct quantitative insight into the dissociation dynamics of SpA-hIgG1 complex and probe the role of the hot spots of SpA. Therefore, CG-MD simulations coupled with the Martini force field is an effective method to study the dissociation dynamics of the protein-protein complex.

## Supporting Information

Figure S1 **Inter-molecular potential energy contribution of some residues of the SpA-hIgG1 complex in 0.25 mol/L NaCl solution at pH 7.0.** (a) SpA and (b) hIgG1.
(TIF)

Figure S2 **Inter-molecular potential energy contribution of some residues of the SpA-hIgG1 complex in 0.5 mol/L NaCl solution at pH 7.0.** (a) SpA and (b) hIgG1.
(TIF)

Figure S3 **Inter-molecular potential energy contribution of some residues of the SpA-hIgG1 complex in 1.0 mol/L NaCl solution at pH 7.0.** (a) SpA and (b) hIgG1.
(TIF)
