# Supplementary figures and images for: Molecular Basis for the Dissociation Dynamics of Protein A-Immunoglobulin G1 Complex
Source: PLoS One. 2013 Jun 12;8(6):e66935. doi: 10.1371/journal.pone.0066935 (PMC3680412; doi:10.1371/journal.pone.0066935)

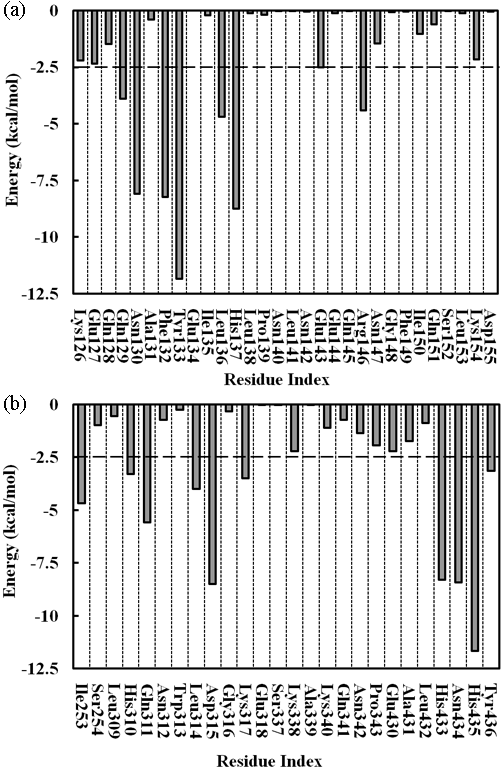

Supplement: Figure S1 — Inter-molecular potential energy contribution of some residues of the SpA-hIgG1 complex in 0.25 mol/L NaCl solution at pH 7.0. (a) SpA and (b) hIgG1. (TIF) [file pone.0066935.s001.tif]

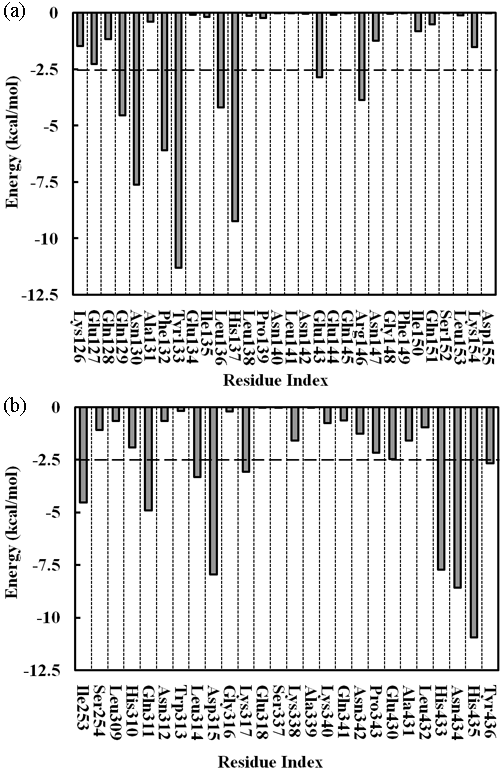

Supplement: Figure S2 — Inter-molecular potential energy contribution of some residues of the SpA-hIgG1 complex in 0.5 mol/L NaCl solution at pH 7.0. (a) SpA and (b) hIgG1. (TIF) [file pone.0066935.s002.tif]

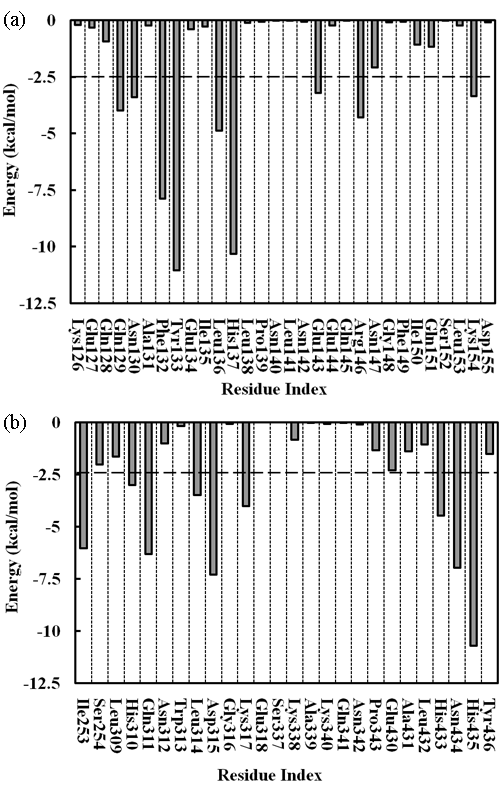

Supplement: Figure S3 — Inter-molecular potential energy contribution of some residues of the SpA-hIgG1 complex in 1.0 mol/L NaCl solution at pH 7.0. (a) SpA and (b) hIgG1. (TIF) [file pone.0066935.s003.tif]
